# Effect of post-discharge virtual wards on improving outcomes in heart failure and non-heart failure populations: A systematic review and meta-analysis

**DOI:** 10.1371/journal.pone.0196114

**Published:** 2018-04-30

**Authors:** Kelsey Uminski, Paul Komenda, Reid Whitlock, Thomas Ferguson, Stewart Nadurak, Laura Hochheim, Navdeep Tangri, Claudio Rigatto

**Affiliations:** 1 Max Rady College of Medicine, University of Manitoba, Winnipeg, Canada; 2 Chronic Disease Innovation Centre, Seven Oaks General Hospital, Winnipeg, Canada; 3 Department of Community Health Sciences, University of Manitoba, Winnipeg, Canada; 4 Department of Library Services, University of Manitoba, Winnipeg, Canada; Osaka University Graduate School of Medicine, JAPAN

## Abstract

**Background:**

Unplanned hospital admissions in high-risk patients are common and costly in an increasingly frail chronic disease population. Virtual Wards (VW) are an emerging concept to improve outcomes in these patients.

**Purpose:**

To evaluate the effect of post-discharge VWs, as an alternative to usual community based care, on hospital readmissions and mortality among heart failure and non-heart failure populations.

**Data sources:**

Ovid MEDLINE, EMBASE, PubMed, the Cochrane Database of Systematic Reviews, SCOPUS and CINAHL, from inception through to Jan 31, 2017; unpublished data, prior systematic reviews; reference lists.

**Study selection:**

Randomized trials of post-discharge VW versus community based, usual care that reported all-cause hospital readmission and mortality outcomes.

**Data extraction:**

Data were reviewed for inclusion and independently extracted by two reviewers. Risk of bias was assessed using the Cochrane Collaboration risk of bias tool.

**Data synthesis:**

In patients with heart failure, a post-discharge VW reduced risk of mortality (six trials, n = 1634; RR 0.59, 95% CI = 0.44–0.78). Heart failure related readmissions were reduced (RR 0.61, 95% CI = 0.49–0.76), although all-cause readmission was not. In contrast, a post-discharge VW did not reduce death or hospital readmissions for patients with undifferentiated high-risk chronic diseases (four trials, n = .3186).

**Limitations:**

Heterogeneity with respect to intervention and comparator, lacking consistent descriptions and utilization of standardized nomenclature for VW. Some trials had methodologic shortcomings and relatively small study populations.

**Conclusions:**

A post-discharge VW can provide added benefits to usual community based care to reduce all-cause mortality and heart failure-related hospital admissions among patients with heart failure. Further research is needed to evaluate the utility of VWs in other chronic disease settings.

## Introduction

Unplanned hospital admissions are common and costly [1]. Following discharge, over one third of patients are readmitted within 90 days, contributing to the estimated 17.4 billion dollar annual cost to Medicare for readmissions [2]. These costs are similarly high in other healthcare systems [3]. Furthermore, in the United States, many health centers are held financially accountable by payers for readmissions deemed avoidable [4]. Patients particularly vulnerable to unplanned hospital readmissions are those with multiple chronic conditions and complex care needs [5–6]. Upwards of 59% of hospital readmissions may be avoidable [7]. This has led many to evaluate alternative strategies to improve the integration of healthcare for patients at high risk of future hospitalizations [8].

The transition from hospital to home is a period of vulnerability in the patient journey [9–10], in which patients and families struggle to assume management of their own care [11–13]. To improve the continuity of care for high-risk patient populations post-discharge and mitigate the risk of hospital readmissions, the virtual ward (VW) model is conceptually appealing. The VW provides patients with a period of intensive multidisciplinary team management, employing the “systems, staffing, and daily routines of a hospital ward…” in a community-based care framework [14]. In addition to a multidisciplinary healthcare team, VW models frequently incorporate telehealth and case management components, representing a much higher level of care team integration than is typical of case management or telehealth interventions alone [14]. The VW model is distinct from the hospital at home scheme, where patients who would otherwise require admission to hospital are instead provided with acute hospital care in the home [15].

There is evidence that VWs may be effective in reducing hospital readmissions and may improve short-term survival for certain chronic conditions such as heart failure [16–17]. However, other studies examining a more general hospital population have found conflicting results [18–20]. In order to inform an optimal utilization for VWs in the management of common conditions that require hospitalization, we performed a systematic review to identify relevant studies assessing the efficacy of a post-discharge VW on hospital readmissions and mortality in high-risk heart failure and non-heart failure populations. Our primary focus was on populations with congestive heart failure, chronic kidney disease, chronic obstructive pulmonary disease and high-risk medical conditions.

## Materials and methods

Our systematic review was performed with a pre-specified protocol and is reported in accordance with the Preferred Reporting Items for Systematic Reviews and Meta-analyses (PRISMA) Statement [21]. We did not register this protocol with traditional systematic review registries (eg. PROSPERO).

### Definition of virtual ward

We identified 4 operational a priori criteria to distinguish a VW from less intensive telemonitoring and case management interventions: 1) The care provided is similar to that provided by an interdisciplinary hospital ward team, 2) Care is longitudinally coordinated by an interdisciplinary team comprising at least two health professionals (e.g. MD, Nurse); 3) Care may be delivered in the patient’s home, through telephone or at a local clinic; 4) Care can include telemonitoring and case managers; however, there must be clear and evident oversight and integration of patient care by the interdisciplinary team. These criteria were designed to capture the higher intensity and integration of care characteristic of a VW compared to telemonitoring or case management, congruent with the core definition of a VW [13]. All 4 criteria had to be met to classify an intervention as a VW. Interventions that did not utilize the term “virtual ward”, but met our four operational criteria, were included in our review.

### Data sources and searches

We searched the following databases from the date of their establishment through to 31 Jan 2017: MEDLINE, EMBASE, PubMed, the Cochrane Database of Systematic Reviews, SCOPUS and CINAHL. Specific search strategies were developed for each database in consultation with a medical librarian. The search strategy was tailored to each database using a combination of MeSH and keywords to cover the concepts of “virtual ward”, “chronic disease” and “high risk patients” (S1 Text). We supplemented these electronic searches with a manual search of the reference lists of included studies and a recent review [8].

### Study selection

Patients of interest were community dwelling adults (aged 18+) recruited immediately or up to three months post-discharge to a VW intervention. We included only prospective, randomized controlled trials. No language restrictions were applied. Titles and abstracts of all citations identified in the search were independently assessed by two reviewers for potential study inclusion. If either reviewer considered the citation potentially relevant, the full-text article was retrieved for further independent evaluation by each of the two reviewers. After independent full-text review, articles for which there was unanimous agreement inclusion were selected for data extraction. In the case of unanimously excluded articles, reason(s) for study exclusion were documented. Disagreements about articles were resolved by consensus, and if unresolved, a third reviewer was consulted.

### Data extraction

Data were collected independently by two reviewers, with inconsistencies resolved by consensus. The following information was extracted from each study: first author, journal, year of publication, study location, study population, mean age, proportion of females, study design, including elements related to risk of bias as described below, sample size, description of VW and usual care, duration of intervention and observation, absolute rate of hospital readmissions and mortality in intervention versus control arms with associated confidence intervals.

### Quality assessment

We evaluated risk of bias through application of the Cochrane Collaboration’s Tool (CCT) for assessing risk of bias in randomized studies [22–23]. The CCT allows for the classification of trials into low-, unclear-, or high-risk categories based on possible risks to study validity stemming from selection bias, performance bias, detection bias, attrition bias, reporting bias, and other biases. The risk of bias assessment was independently performed by two reviewers, with disagreements resolved by consensus.

### Data synthesis and analysis

The ten studies meeting our inclusion criteria naturally clustered themselves into two groups. The first group was composed of studies exclusively in heart failure patients, and the other, studies in undifferentiated high-risk chronic disease patients. For each group, we used random effects meta-analyses to estimate pooled risk ratios and 95% confidence intervals for mortality and hospital readmissions attributable to the VW intervention vs. usual care. Heterogeneity among studies for these outcomes was assessed using both the Cochran’s Q test [24] and the I^2^ statistic [25]. Heterogeneity by way of the I^2^ statistic was classified as low, medium and high based on benchmarks of 25%, 50% and 75% respectively [26]. We performed sensitivity analyses to assess the impact on the results of 1) removing studies at high risk of bias and 2) examining only 30-day readmission rates. We also examined the impact of case mix on the efficacy of VW in those studies considering an undifferentiated high-risk chronic disease population.

## Results

### Study selection

**Fig 1:**
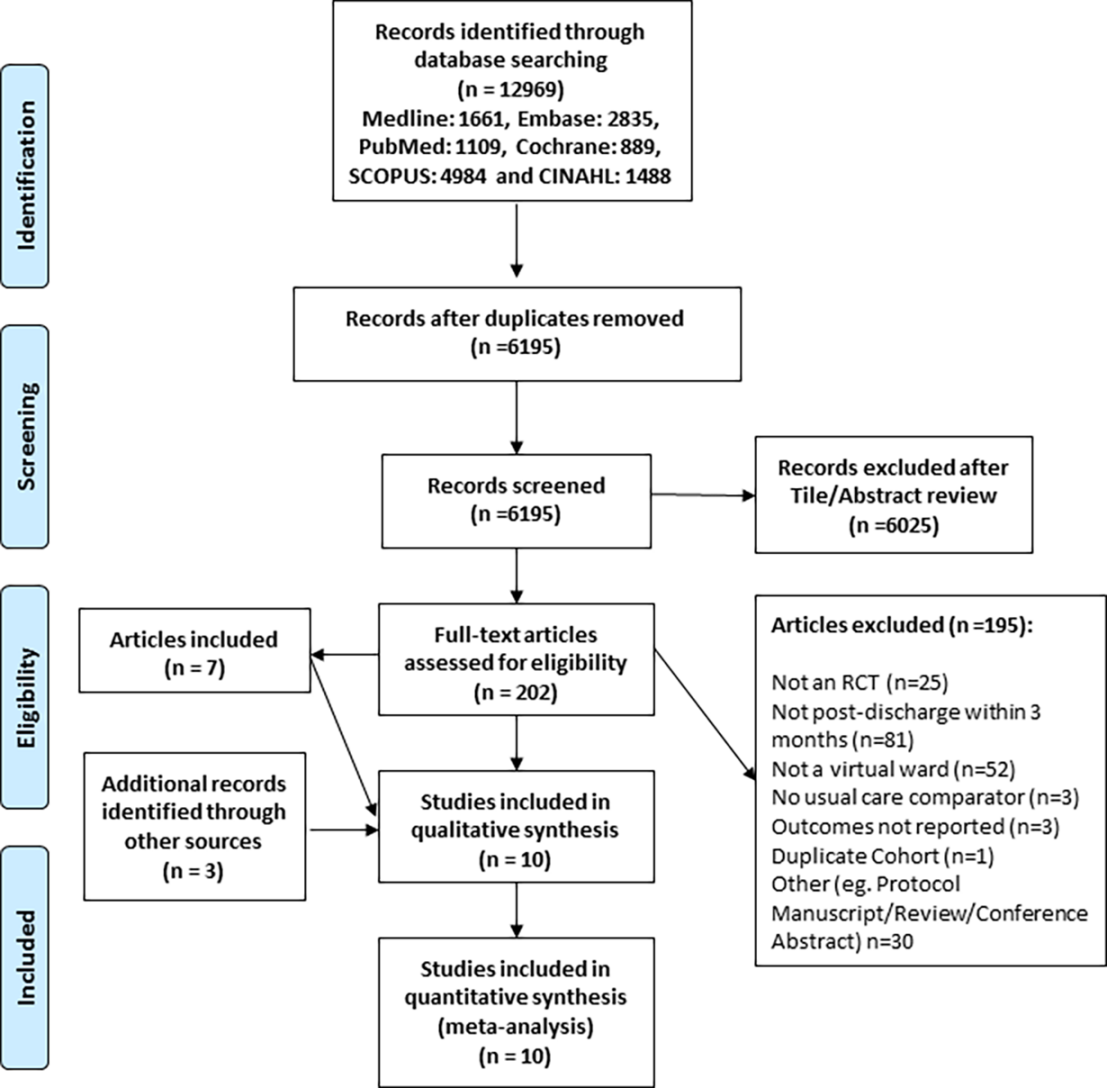
Flow diagram for trial selection and exclusion.

A flow diagram outlining the selection strategy is shown in Fig 1. Our search strategy yielded 6025 studies for screening. Of these, 202 studies were selected for full text review, and seven met the inclusion criteria for our systematic review. Screening the reference lists of included studies and a recent review [8] identified three additional studies for inclusion (Fig 1).

### Characteristics of selected studies

The included trials [17, 19, 27–34] randomized a total of 4820 patients. Of the ten included studies, six were in patients with heart failure exclusively, while the remaining four comprised an undifferentiated high-risk chronic disease group. No randomized trials meeting the VW definition exclusively examined chronic kidney disease or chronic obstructive pulmonary disease populations. The period of intervention ranged widely, from 30 days to one year. Each of the study interventions comprised a multidisciplinary team, the majority of which also included the patient’s general practitioner. For each of the studies, patient contact was conducted at a minimum bi-weekly, but most often weekly. Telemonitoring was a component of the VW intervention in most studies examining heart failure patients, which was generally nurse-led, with heart failure specialist support. Structured communication amongst each of the multidisciplinary care providers was a requisite for the VW intervention; however, several studies expanded on this concept by designing and implementing an online database to do so.

For each of the included studies, the control intervention was comprised of post-discharge outpatient follow-up, more often with the patient’s primary care physician; however, in several studies, structured follow-up care with the patient’s cardiologist was arranged for further management. Patient post-discharge management was informed by a discharge summary provided to community based care providers following hospitalization. In only one of the included studies, follow-up care was exclusively through the management of a congestive heart-failure team clinic, and in another follow-up care was not explicitly outlined. A table outlining the characteristics of the selected studies is shown in Table 1.

**Table 1:** Overview of study characteristics.

First Author, YR	Country	N	Period of Intervention	Description of Usual Care	Description of Virtual Ward Intervention	Patient Population	Mean Age	% Female
***Heart Failure Patient Population***								
**Angermann, 2012**	Germany	715	6 months	Patients received standard post-discharge planning encompassing therapeutic plans, discharge letters, and scheduled follow-up appointments with either a general practitioner or cardiologist in 1–2 weeks. No restrictions were imposed on outpatient care.	In addition to usual care, patient telemonitoring and education involving nurses, general practitioners and cardiologists. Nurse-driven telephone contacts for cardiac monitoring and inquiries into general health informed patient care plans.	Patients aged 18+, hospitalized with decompensated heart failure, and a LVEF ≤40%	69	29
**Antonicelli, 2008**	Italy	57	12 months	Patients received follow-up care in the form of routinely scheduled visits every four months, with additional visits as deemed necessary, with a team specialized in heart failure management. Patients were contacted on a monthly basis to collect data on new hospital admissions, cardiovascular complications and death. Discharge counseling regarding therapeutic medication and lifestyle adherence was provided.	Patient telemonitoring involving specialized heart failure team. Team-driven telephone contacts for cardiac monitoring, medication adherence, and inquiries into general health conducted at least once weekly in addition to a weekly EKG transmission. Therapeutic regimen was regularly reassessed and altered when necessary. Clinic visits were performed whenever necessary, based on telemonitored data or telephone interviews.	Patients aged 70+, hospitalized with decompensated heart failure	78	39
**Dendale, 2012**	Belgium	160	6 months	Patients received a nurse-led heart failure education course before discharge. Follow-up care was arranged at two-weeks post-discharge in the heart-failure clinic for patient assessment and treatment modifications, if necessary. Subsequent management was under the care of the patient's general practitioner who could refer the patient for cardiology management if needed.	In addition to usual care patients were seen in the outpatient heart failure clinic with additional planned visits at 3 and 6 months. Daily patient telemonitoring was conducted with specified alert limits set for each patient. Alterations in patient status were forwarded to the general practitioner and heart failure clinic for subsequent patient follow-up and management. Following changes to therapeutic regimen, a nurse-led telephone follow-up assessed intervention efficacy. General practitioners were free to contact the patient as desired. An online database was created to facilitate cross-communication between the general practitioner and heart failure team, to optimize patient management.	Patients hospitalized with decompensated heart failure. All patients had to be treated with an angiotensin-converting enzyme inhibitor or angiotensin receptor blocker, and a beta-blocker in the absence of contraindications.	76	35
**Giordano, 2009**	Italy	460	12 months	Patients received structured follow-up care from their primary care physician within two weeks of discharge in addition to an appointment with their cardiologist at 12 months post-discharge for assessment.	Patient telemonitoring involving medical and nursing professionals. Daily transmission of cardiac parameters was monitored by a cardiologist, general practitioner and nurse, who assessed the patient’s clinical status, providing consultation or triage. Nurse-driven telephone contacts to assess patient status and treatment regimen adherence were conducted weekly, or bi-weekly, dependent on patient status. Patient clinical appointments and additional investigations were requested as per patient status. Once a week the cardiologist and the nurse met together to sum up a clinical course of the enrolled patients. An online database was created as a patient record, to optimize patient management.	Patients hospitalized with decompensated heart failure, LVEF<40% and at least one hospitalization for acute heart failure in the previous year.	57	15
**Kasper, 2002**	USA	200	6 months	Patients received unrestricted follow-up care from their primary physicians, who received a baseline heart failure management plan, as documented in the patient's chart.	Patients received nurse-led care coordination linked to a multidisciplinary team composed of a heart failure nurse, cardiologist and patient’s primary care physician. Patients were contacted via telephone at preplanned intervals after discharge, in addition to scheduled visits within the community. Depending on patient factors, visits could be scheduled more frequently. Weekly care team meetings were held to discuss and optimize patient management.	Patients hospitalized with decompensated heart failure, NYHA Class III/IV, and presence of one or more additional designated high-risk criteria: aged 75+, LVEF<35%, one additional heart failure admission in past year, ischemic cardiomyopathy, peripheral edema at hospital discharge, <3kg of weight loss during hospital stay, peripheral vascular disease, pulmonary capillary wedge pressure >25mmHg, cardiac Index <2.0L/min/m2, systolic blood pressure >180mmHg or diastolic blood pressure >100mmHg.	62	39
**Leventhal, 2011**	Switzerland	42	12 months	Patients received unrestricted follow-up care from their primary care physician.	Patients received structured telephone support/assessments and home visits led by a heart failure nurse specialist. A multidisciplinary approach was taken to individualize care plans with the inclusion of the patient’s primary care physician, and internist, cardiology and dietary consultation where necessary.	Patients hospitalized with decompensated heart failure, NYHA Class II-IV, irrespective of LVEF, and aBNP≥ 100pg/mL. Additional criteria included history of dyspnea, increased fatigue or weakness, and German speakers.	77	38
***Undifferentiated High Risk Chronic Disease Patients***								
**Caplan, 2004**	Australia	370	30 days	Patients were discharged to home with a therapeutic management plan as outlined by the emergency department medical officer.	Patients received nurse-led care coordination in addition to care management from a multidisciplinary team. A nurse-led home visit was conducted within 24 hours of discharge. Information collected on patient status was subsequently used to formulate a care plan, and initiate interventions and referral. Weekly interdisciplinary team meetings, composed of a geriatrician or a geriatric registrar, nurses, physiotherapists, and occupational therapists, were held to discuss optimization of patient care plans. Throughout the process, the nurse care coordinator liaised with the patient's general practitioner.	Patients aged years 75+, discharged from the emergency department.	82	60
**Dhalla, 2014**	Canada	1923	> 12 months	Patients and their primary care providers received a discharge summary, a therapeutic plan including prescriptions and home care arrangements as necessary. Patients received discharge counseling from a member of the health care team. Patients received either recommended or scheduled appointments for follow-up care with their primary care or specialist physicians. While not routine, follow-up care within the hospital's post-discharge clinic could be arranged by the discharging hospital physician as seen fit.	Usual care plus, patients received care coordination in addition to care management from a multidisciplinary team through telephone, home visits, and/or clinic visits. The VW team consisted of care coordinators, a pharmacist, a nurse or nurse practitioner, a physician, and a clerical assistant. Daily meetings were held with the team to discuss enrolled patients and design/modify individual treatment plans.	Patients aged years 18+, discharged from a general internal medicine ward, and LACE score ≥10.	71	49
**Hansen, 1995**	Denmark	193	6 months	Patients received follow-up care from their general practitioner, who received a discharge summary. Unspecified social supports were provided to patients on the day of patient discharge. No geriatric follow-up visits within the community were provided.	A multidisciplinary team composed of a geriatrician, nurse and physical therapist conducted multiple scheduled patient visits, with additional visits informed by patient need. Serial patient evaluations informed patient management strategies and need for optimization of care plans. Communication with the patient’s general practitioner was maintained over the course of the intervention.	Patients discharged from a subacute geriatric ward	80	67
**Rytter, 2010**	Denmark	333	12 weeks	Patients received follow-up care from their general practitioner who received post-discharge letters.	Patient follow-up consisted of three contacts: a joint home visit involving both a GP and nurse at one-week, with either clinic or home-visit at three and eight weeks post-discharge. At each visit patient care plans were reevaluated and changes according to patient status implemented.	Patients aged years 78+, discharged from the geriatric or internal medicine wards, and hospitalized for a minimum of two days.	83	66

### Quality of reporting and risk of bias of the included studies

The risk of bias for the included studies is summarized individually and in aggregate (S1 and S2 Figs). Of the ten included studies, three studies had low risk of selection bias, incorporating random sequence generation with allocation concealment. Given the nature of the intervention, blinding was not possible for participants and most study personnel. Only three studies described blinding of the outcomes assessment, however. Six studies reported an intention-to-treat analysis. All pre-specified outcomes were reported in every study, indicating a low risk of reporting bias. Overall, three of the ten studies were felt to be at high risk of bias [28, 32, 33].

### Analysis of studies considering heart failure patients

**Fig 2:**
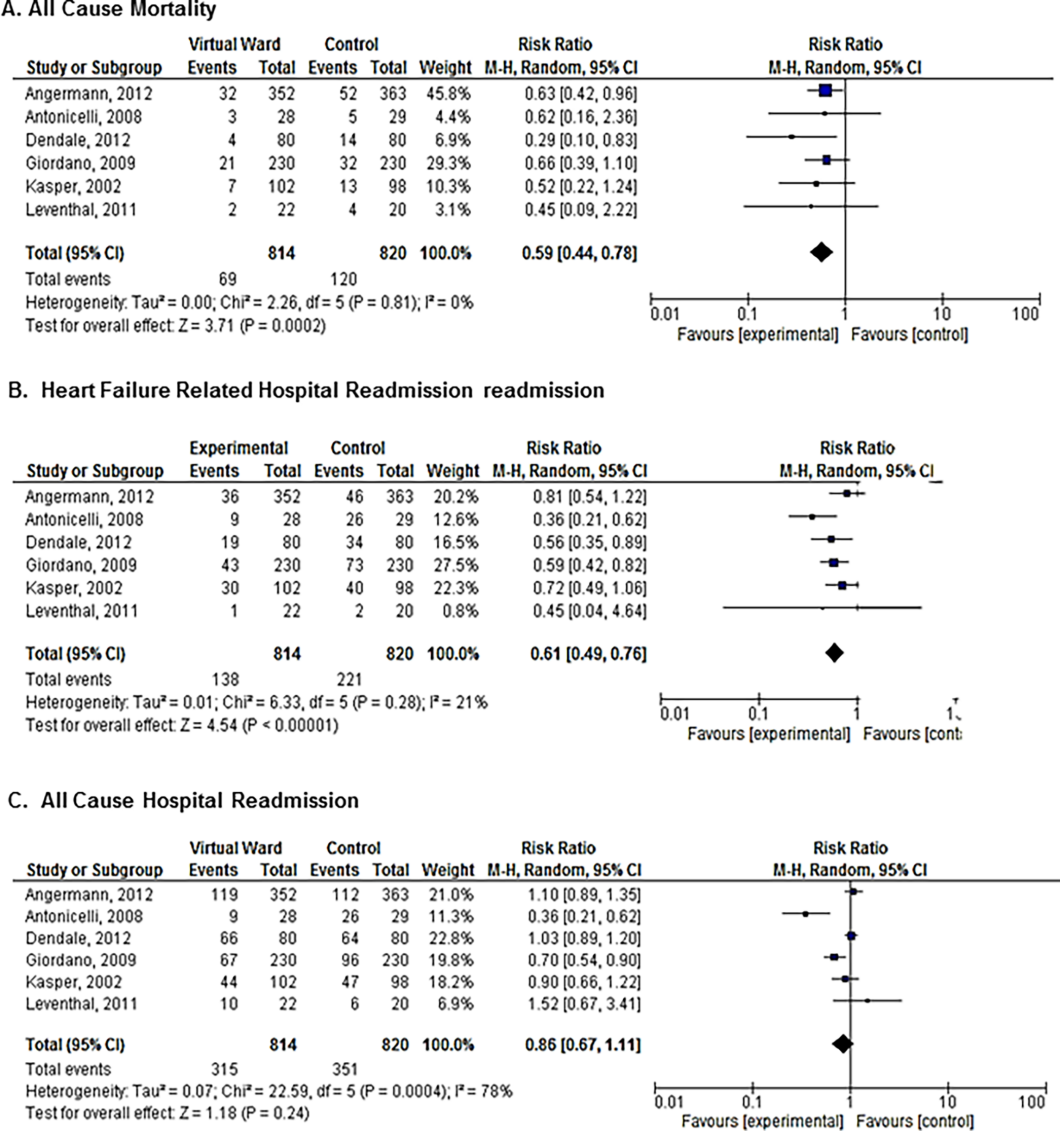
Meta-analysis of the relative risks of mortality and hospital readmission in studies of heart failure patients: (A) All-cause mortality; (B) Heart failure related hospital readmission; (C) All cause hospital readmission.

Six studies (n = 1634) examined the efficacy of a VW compared to usual care in patients with heart failure. The VW was associated with a reduced risk of mortality (RR 0.59, 95% CI = 0.44–0.78; (I^2^ = 0%); Fig 2A) and cause-specific hospital readmission (RR 0.61, 95% CI = 0.49–0.76; I^2^ = 21%; Fig 2B). VW did not reduce all-cause hospital admissions (composite RR 0.86, 95% CI = 0.67–1.11; I^2^ = 0%; Fig 2C).

### Analysis of studies considering undifferentiated high-risk chronic disease patients

**Fig 3:**
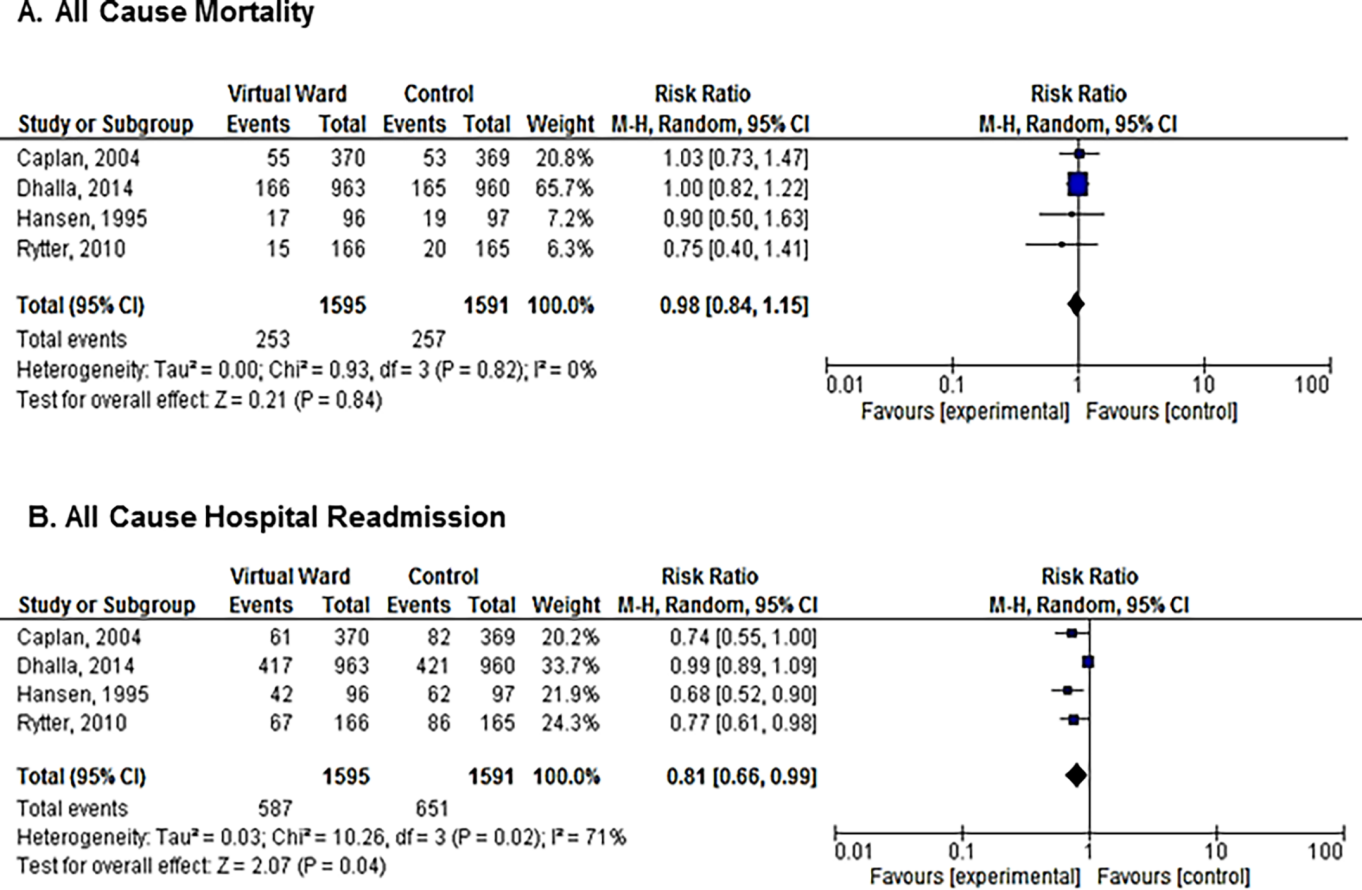
Meta-analysis of the relative risk of mortality and hospital readmission in studies of undifferentiated chronic disease patients: (A) All-cause mortality; (B) All cause hospital readmission.

Four studies examined the efficacy of a VW in undifferentiated high-risk chronic disease patients (n = 3186). Heterogeneity among studies was low for the outcome of mortality (I^2^ = 0%). VW’s did not reduce mortality (RR 0.98, 95% CI = 0.84–1.15; Fig 3A) compared to usual care in this undifferentiated high-risk chronic disease population. There appeared to be a reduction in hospital readmissions with VWs under a random effects model (RR 0.81, 95% CI = 0.66–0.99; Fig 3B); however, there was considerable heterogeneity for this outcome (I^2^ = 71%).

### Sensitivity analyses

#### Exclusion of studies at highest risk of bias

Excluding the studies at highest risk of bias did not alter the composite risk ratios, all of which were similar in magnitude and direction to the main analysis (S3–S6 Figs).

#### Impact of follow-up time on efficacy for hospital readmissions

Because variability in follow-up time among studies could have increased the among-study heterogeneity for the outcome of hospital readmission, we tried to eliminate this source of variation by contacting study authors to obtain 30 day readmission rates for all studies. We successfully obtained 30-day readmission data in four of six heart failure studies and three of four undifferentiated high risk chronic disease studies. Heterogeneity was indeed reduced when 30-day readmission data were used. However, the composite relative risks were similar to the main analyses in both heart failure and undifferentiated high-risk chronic disease groups (S7 and S8 Figs).

#### Impact of case mix on efficacy of VW in studies of undifferentiated high-risk patients

We examined whether the proportion of patients with a primary cardiac admission diagnosis influenced the estimate of VW efficacy in studies of undifferentiated high-risk medical patients (S9 Fig). There was a trend towards lower relative risk of hospital readmission with the VW intervention in studies with higher proportion of patients admitted for cardiac reasons.

## Discussion

In this systematic review and meta-analysis evaluating the efficacy of a post-discharge VW, our main finding was that a VW model of care applied after discharge from hospital reduced all-cause mortality and heart failure-related hospital readmissions in patients with congestive heart failure. There was no effect on all-cause hospital readmissions in heart failure patients. There was no effect in an undifferentiated high-risk chronic disease population. These findings suggest that disease specificity of the VW intervention appears to be important.

To our knowledge, our systematic review provides the first and largest assessment of post-discharge VWs as a pre-emptive health intervention to improve post discharge outcomes in high-risk chronic disease groups, and includes most of the contemporary studies to date. Other systematic reviews have addressed similar but conceptually distinct interventions such as hospital at home [14], case management [35], and telemonitoring [36]. Hospital at home interventions are similar to VW’s in that multifaceted, highly structured and interdisciplinary care is provided at home, but differ in terms of purpose and target patient group. Hospital at home is designed to replace the care normally provided in hospital for acute or acute on chronic conditions, is targeted to patients being considered for hospital admission from clinics or the emergency department, and constitutes an alternative treatment path to hospital admission. In contrast, VWs are targeted to patients being discharged from hospital, and the primary purpose of the care is to help patients transition from hospital to home. Case management and telemonitoring interventions can also be used to address this transition, but typically are narrower in scope and simpler in design than VWs, which are distinguished by a higher degree of interdisciplinary review and coordination. VWs often incorporate telehealth and case management features, but these aspects are integrated into interdisciplinary teams that regularly and virtually “round” on patients in a way similar to a team of doctors, nurses and allied health professionals in a hospital medical ward [9]. Specialized disease management programs utilizing structured telephone support and telemonitoring have been evaluated and employed in a number of chronic disease conditions [37]. A recent overview of systematic reviews, synthesizing the results of 15 studies in heart failure patients concluded that telephone support and telemonitoring as a component of patient management reduces all-cause patient mortality and disease related-hospital admissions [36]. Similarly, heart failure patients who receive case management interventions have been shown to have less all-cause mortality and are less likely to be readmitted to hospital one year following discharge [35]. Our observations on the effects of VW in heart failure are congruent with these findings.

Our results further suggest that a specific, disease-focused intervention (e.g. as in studies of VW’s in heart failure) may be more effective at reducing mortality than broader VW interventions (e.g. VW’s in undifferentiated high-risk medical populations). Although the reasons for this are incompletely understood, it seems plausible that characteristics of both the intervention and the target population play a role. It is likely that a relatively simple VW, narrowly focused on a small set of key disease modifying interventions (e.g. diuretic titration according to daily weights and blood pressures in heart failure patients), and applied in a relatively homogeneous population with a single dominant disease (e.g. heart failure), may be more effective at preventing death or re-hospitalization than a more complex VW targeting patients with multiple comorbid conditions. Such patients may be at risk from multiple competing pathways for death, and consequently no discrete bundle of interventions may be able to maintain health.

It is noteworthy that VW reduced mortality and heart failure readmissions, but not all cause readmissions, in heart failure studies. In theory, a VW could influence heart failure patient survival in 3 ways: 1) prevention of worsening heart failure, 2) improved early identification and treatment of life-threatening heart failure-related complications or 3) improved identification and treatment of life-threatening non-heart failure complications (e.g. myocardial infarction, arrhythmia, stroke, bleeding). Any of the three pathways, if valid, could reduce mortality. Pathway 1 would, in addition, be expected to reduce admissions for heart failure. Pathways 2 and 3, however, would not necessarily reduce admissions, particularly if the improved mortality outcome were mediated by early identification followed by preemptive admission for treatment. None of these pathways are mutually exclusive, and all might contribute to the overall effect, if true. We found that heart failure-related readmissions were substantially and consistently reduced, congruent with pathway 1 and a heart failure treatment related reduction in mortality[27].

Although we did find a statistically significant reduction in readmissions among studies in undifferentiated high-risk chronic diseases, this finding should be interpreted cautiously as there was pronounced heterogeneity, making interpretation of the composite risk ratio less clear. Moreover, when studies at high risk of bias were excluded, the signal favoring VW was attenuated.

Our review has several strengths. We sought to include all relevant randomized control trials, searched broadly across multiple databases, and used a pre-specified definition of VW, which was objectively and rigorously applied, minimizing study selection bias. We meta-analyzed both mortality and hospital readmissions, as both these outcomes are relevant to patients and caregivers. Our analysis comprised ten studies randomizing over 4800 patients.

Our review also has limitations. First, as community-based pre-emptive care is an evolving field, terminology is not standardized [13, 38]. The term “virtual ward” has not been uniformly adopted, making identification of relevant studies challenging. We addressed this problem by 1) searching broadly, using multiple conceptually related terms (S1 Text) and then 2) applying a pre-specified operational definition for VW, based on the original concept and definition. Second, variability across studies in the structure of the intervention or control groups was evident and likely contributed to statistical heterogeneity. Despite this, we found little statistical heterogeneity for the outcomes of all-cause mortality and heart failure-related readmissions, increasing our confidence in these findings. On the other hand, heterogeneity was high for all cause readmission among studies examining an undifferentiated high-risk chronic disease population, necessitating a more cautious interpretation. Third, descriptions of the VW component interventions were not sufficiently granular to allow analysis of the relative importance of each component to the impact of the intervention. Fourth, publication bias is always a potential limitation in any review; however, the fact that we found no evidence for efficacy for VWs in non-heart failure populations argues against strong publication bias in favor of positive trials. Bias within studies is also a consideration; reassuringly a sensitivity analysis excluding studies at high risk of bias did not alter the findings (S3 to S6 Figs). Finally, the role of intervention fidelity was, and the cost-effectiveness of VW compared to usual care, were not reported in the included studies.

Despite these limitations, we believe our findings provide a valid summary of evidence to date, and as such have implications for research and clinical care. A post-discharge VW appears to improve all-cause mortality and heart failure-related hospital readmissions in patients admitted for heart failure. Future efforts should focus on streamlining implementation and improving cost-effectiveness in this patient group. It seems likely that disease-specific VWs will be more effective than general interventions addressing a heterogeneous high-risk population; future studies of VWs should probably focus on other homogeneous chronic disease populations such as chronic obstructive pulmonary disease and chronic kidney disease. Finally, greater standardization and consistency in the use of the term “virtual ward” would help distinguish this intervention from case management, telemonitoring, and other transitional care interventions, facilitating future knowledge synthesis and translation.

## Conclusions

Compared to usual community based care, a post-discharge VW can reduce mortality and disease specific hospital readmissions in patients with heart failure, but not in more heterogeneous high-risk populations. We conclude that disease specificity of the VW intervention appears to be important. Further research is needed to assess the utility of virtual wards in other chronic disease groups.

## Supporting information

S1 Table PRISMA 2009 checklist. (DOC)

S2 Table Virtual full text review updated with SCOPUS search. (XLSX)

S1 Fig Risk of bias assessment of included studies using the Cochrane Collaboration’s Tool for randomized studies. (DOC)

S2 Fig Risk of bias assessment for all included studies stratified according to each of the Cochrane Collaboration’s Tool for randomized studies criteria. (DOC)

S3 Fig Meta-analysis of the relative risk of all-cause mortality in studies in heart failure populations, excluding those studies deemed at high risk of bias. (DOC)

S4 Fig Meta-analysis of the relative risk of heart-failure related hospital readmission in studies in heart failure populations, excluding those studies deemed at high risk of bias. (DOC)

S5 Fig Meta-analysis of the relative risk of all-cause mortality in studies in undifferentiated high-risk chronic disease patients, excluding those studies deemed at high risk of bias. (DOC)

S6 Fig Meta-analysis of the relative risk of all-cause hospital readmission in studies in undifferentiated high-risk chronic disease patients, excluding those studies deemed at high risk of bias. (DOC)

S7 Fig Sensitivity analysis using 30-day admission rates in studies of heart failure patients. (DOC)

S8 Fig Sensitivity analysis using 30-day admission rates in studies of undifferentiated high risk patients. (DOC)

S9 Fig Relative risk of hospital admissions as a function of proportion of cardiac patients comprising the undifferentiated high-risk chronic disease group. (DOC)

S1 Text Search strategies. (DOC)

## Abbreviations

VW: Virtual Ward
CCT: Cochrane Collaboration’s Tool for assessing risk of bias in randomized studies
