# Supplementary material for: Effect of post-discharge virtual wards on improving outcomes in heart failure and non-heart failure populations: A systematic review and meta-analysis
Source: PLoS One. 2018 Apr 30;13(4):e0196114. doi: 10.1371/journal.pone.0196114 (PMC5927407; doi:10.1371/journal.pone.0196114)
Supplement: S4 Fig — (DOC) [file pone.0196114.s006.doc]

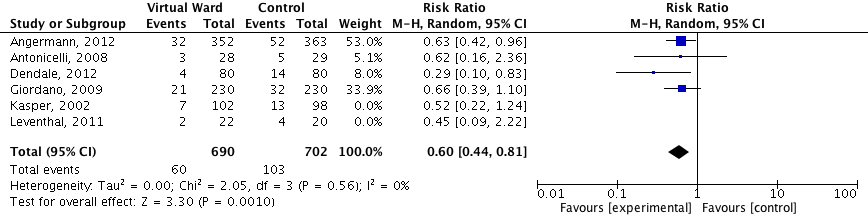


**S4 Fig**. **Meta-analysis of the relative risk of heart-failure related hospital readmission in studies in heart failure populations, excluding those studies deemed at high risk of bias.**
