# Supplementary material for: Effect of post-discharge virtual wards on improving outcomes in heart failure and non-heart failure populations: A systematic review and meta-analysis
Source: PLoS One. 2018 Apr 30;13(4):e0196114. doi: 10.1371/journal.pone.0196114 (PMC5927407; doi:10.1371/journal.pone.0196114)
Supplement: S8 Fig — (DOC) [file pone.0196114.s010.doc]

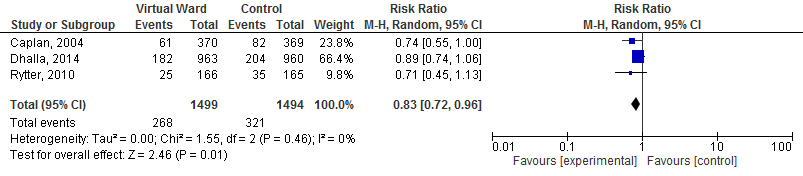


**S8 Fig.** **Sensitivity analysis using 30-day admission rates in studies of undifferentiated high risk patients**
