# Supplementary material for: Effect of post-discharge virtual wards on improving outcomes in heart failure and non-heart failure populations: A systematic review and meta-analysis
Source: PLoS One. 2018 Apr 30;13(4):e0196114. doi: 10.1371/journal.pone.0196114 (PMC5927407; doi:10.1371/journal.pone.0196114)
Supplement: S9 Fig — (DOC) [file pone.0196114.s011.doc]

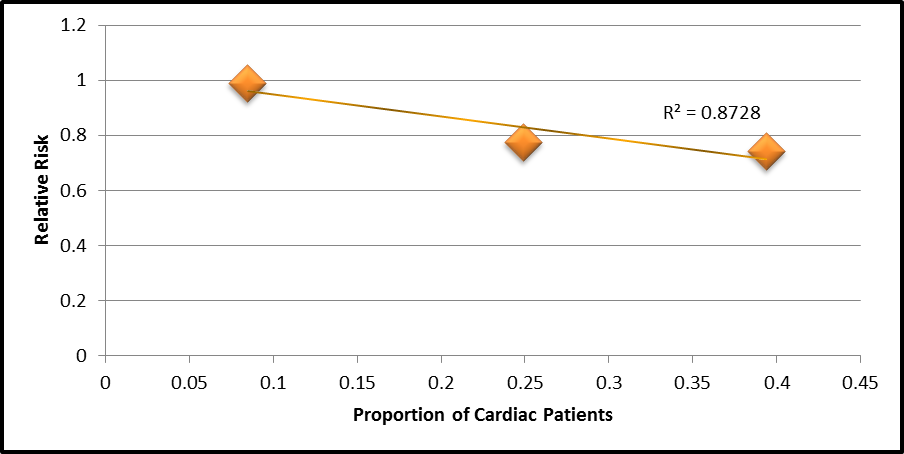


**S9 Fig.** **Relative risk of hospital admissions as a function of proportion of cardiac patients comprising the undifferentiated high-risk chronic disease group.**
