# Supplementary material for: Effect of post-discharge virtual wards on improving outcomes in heart failure and non-heart failure populations: A systematic review and meta-analysis
Source: PLoS One. 2018 Apr 30;13(4):e0196114. doi: 10.1371/journal.pone.0196114 (PMC5927407; doi:10.1371/journal.pone.0196114)
Supplement: S1 Text — (DOC) [file pone.0196114.s012.doc]

**S1 Text.** **Search Strategies**

MEDLINE

1. exp Renal Insufficiency/

2. renal insufficienc$.ti,ab,kw,kf.

3. exp renal replacement therapy/

4. (renal adj2 dialys?s).ti,ab,kw,kf.

5. (Kidney adj2 dialys?s).ti,ab,kw,kf.

6. peritoneal dialys?s.ti,ab,kw,kf.

7. hemodialys?s.ti,ab,kw,kf.

8. extracorporeal dialys?s.ti,ab,kw,kf.

9. Kidney Diseases/

10. kidney disease$.ti,ab,kw,kf.

11. chronic kidney disease$.ti,ab,kw,kf.

12. Chronic renal disease$.ti,ab,kw,kf.

13. end-stage kidney disease$.ti,ab,kw,kf.

14. kidney failure.ti,ab,kw,kf.

15. renal failure.ti,ab,kw,kf.

16. exp Heart Failure/

17. heart failure.ti,ab,kw,kf.

18. cardiac failure.ti,ab,kw,kf.

19. myocardial failure.ti,ab,kw,kf.

20. exp Pulmonary Disease, Chronic Obstructive/

21. COPD.ti,ab,kw,kf.

22. chronic obstructive pulmonary disease.ti,ab,kw,kf.

23. COAD.ti,ab,kw,kf.

24. chronic airflow obstruct$.ti,ab,kw,kf.

25. chronic obstructive lung disease.ti,ab,kw,kf.

26. exp Diabetes Complications/

27. exp Diabetes Mellitus/

28. exp Diabetes Insipidus/

29. diabet$.ti,ab,kw,kf.

30. Frail Elderly/

31. frail elder$.ti,ab,kw,kf.

32. frail older adult$.ti,ab,kw,kf.

33. Mortality/

34. risk factors/

35. (high adj2 risk).ti,ab,kw,kf.

36. 33 or 34 or 35

37. Patient Readmission/

38. readmission.ti,ab,kw,kf.

39. readmit$.ti,ab,kw,kf.

40. 37 or 38 or 39

41 36 and 40

42. 1 or 2 or 3 or 4 or 5 or 6 or 7 or 8 or 9 or 10 or 11 or 12 or 13 or 14 or 15 or 16 or 17 or 18 or 19 or 20 or 21 or 22 or 23 or 24 or 25 or 26 or 27 or 28 or 29 or 30 or 31 or 32 or 41

43. (virtual adj2 ward*).ti,ab,kw,kf.

44. exp Telemedicine/

45. mobile health.ti,ab,kw,kf.

46. mhealth.ti,ab,kw,kf.

47. (hospital adj2 home).ti,ab,kw,kf.

48. remote consultation/

49. teleconsultation.ti,ab,kw,kf.

50. telehealth.ti,ab,kw,kf.

51. or/43-50

52. Ambulatory care/

53. Patient-Specific Modeling/

54. patient specific modeling.ti,ab,kw,kf.

55. Case Management/

56. Monitoring, Physiologic/

57. physiological monitoring.ti,ab,kw,kf.

58. patient monitoring.ti,ab,kw,kf.

59. Patient Care Team/

60. patient care team$.ti,ab,kw,kf.

61. (interdisciplinary adj2 care).ti,ab,kw,kf.

62. interdisciplinary health.ti,ab,kw,kf.

63. (collaborative adj2 care).ti,ab,kw,kf.

64. (health adj2 team$).ti,ab,kw,kf.

65. healthcare team$.ti,ab,kw,kf.

66. or/52-65

67. user-computer interface/

68. Internet/

69. internet.ti,ab,kw,kf.

70. web.ti,ab,kw,kf.

71. virtual.ti,ab,kw,kf.

72. mobile applications/

73. or/67-72

74. 66 and 73

75. 51 or 74

76. 42 and 75

77. randomized controlled trial.pt.

78. controlled clinical trial.pt.

79. randomized controlled trials.sh.

80. random allocation.sh.

81. double blind method.sh.

82. single-blind method.sh.

83. or/1-6

84. (animals not human).sh.

85. 7 not 8

86. clinical trial.pt.

87. exp Clinical Trial/

88. (clin$ adj25 trial$).ti,ab.

89. ((singl$ or doubl$ or trebl$ or tripl$) adj25 (blind$ or mask$)).ti,ab.

90. placebos.sh.

91. placebo$.ti,ab.

92. random$.ti,ab.

93. research design.sh.

94. or/10-17

95. 18 not 8

96. comparative study.sh.

97. exp evaluation studies/

98. follow up studies.sh.

99. prospective studies.sh.

100. retrospective studies.sh.

101. (control$ or prospectiv$ or retrospectiv$ or volunteer$).ti,ab.

102. or/21-26

103. 27 not 8

104. 9 or 20 or 29

105. 76 and 106

**PUBMED:**

("renal insufficiency, chronic"[MeSH] OR “renal insufficiency”[tw] OR “renal insufficiencies”[tw] OR "chronic kidney disease"[tw] OR "chronic kidney diseases"[tw] OR "chronic renal disease"[tw] OR "chronic renal diseases"[tw] OR "kidney failure"[tw] OR "renal failure"[tw] OR "Renal replacement therapy"[mh] OR hemodiafiltration[tw] OR "renal dialysis"[tw] OR "renal dialyses"[tw] OR "hemodialysis"[tw] OR "haemodialysis"[tw] OR "hemodialyses"[tw] OR "haemodialyses"[tw] OR "extracorporeal dialysis"[tw] OR "extracorporeal dialyses"[tw] OR "end-stage kidney disease"[tw] OR "heart failure"[mh] OR "heart failure"[tw] OR "cardiac failure"[tw] OR "myocardial failure"[tw] OR "congestive heart failure"[tw] OR "diastolic heart failure"[tw] OR "systolic heart failure"[tw] OR "pulmonary disease, chronic obstructive"[MeSH] OR COPD[tw] OR COAD[tw] OR "chronic obstructive pulmonary disease”[tw] OR "chronic airflow obstruction"[tw] OR "chronic obstructive lung disease"[tw] OR "diabetes mellitus"[mh] OR diabet*[tw] OR "diabetes insipidus"[MeSH] OR "frail elderly"[MeSH] OR "frail elderly"[tw] OR "frail elders"[tw] OR "frail older adults"[tw] OR ((mortality[MeSH] OR mortalities[tw] OR "risk factors"[MeSH] OR "high risk”[tw]) AND (“patient readmission"[MeSH] OR "hospital readmission"[tw] OR readmit[tw] OR readmitted[tw] OR readmitting[tw]))) AND ("virtual ward"[tw] OR "virtual wards"[tw] OR telemedicine[MeSH] OR "mobile heath"[tw] OR mhealth[tw] OR telehealth[tw] OR "remote consultation"[MeSH] OR "remote consultation"[tw] OR teleconsultation[tw] OR ((“patient-specific modeling"[MeSH] OR "patient specific computational modeling"[tw] OR "monitoring, physiologic"[MeSH] OR "physiological monitoring"[tw] OR "patient monitoring"[tw] OR "ambulatory care"[MeSH] OR "case management”[MeSH] OR "patient care team"[MeSH] OR "patient care team"[tw] OR "patient care teams"[tw] OR "medical care team”[tw] OR “medical care teams”[tw] OR "interdisciplinary health"[tw] OR "health team”[tw] OR “health teams”[tw] OR "collaborative care"[tw] OR "healthcare team"[tw] OR "healthcare teams"[tw]) AND (“user-computer interface"[MeSH] OR "virtual system”[tw] OR “virtual systems”[tw] OR internet[MeSH] OR internet[tw] OR web[tw]))) AND (“randomized controlled trial"[pt] OR "controlled clinical trial"[pt] OR "randomized controlled trials"[tw] OR "random allocation"[mh] OR "double-blind method"[mh] OR "single-blind method"[mh] OR "clinical trial"[pt] OR "clinical trials as topic"[mh] OR placebo[tiab] OR placebos[MeSH] OR random[tiab] OR randomly[tiab] OR randomized[tiab] OR "research design"[mh] OR "comparative study"[pt] OR "evaluation studies"[pt] OR "follow up studies"[mh] OR "prospective studies"[mh] OR "retrospective studies"[MeSH] OR "drug therapy"[sh] OR trial[ti] OR groups[tiab])

**Cochrane:**

#1 MeSH descriptor: [Renal Insufficiency] explode all trees 4970

#2 renal next insufficienc* 2236

#3 MeSH descriptor: [Renal Replacement Therapy] explode all trees 7958

#4 renal next dialys?s 4645

#5 kidney next dialys?s 20

#6 peritoneal next dialys?s 1520

#7 hemodialys?s 4932

#8 haemodialys?s 1599

#9 extracorporeal next dialys?s 5

#10 MeSH descriptor: [Kidney Diseases] explode all trees 10205

#11 "chronic kidney" next disease* 2194

#12 "chronic renal" next disease* 230

#13 "kidney failure" 6441

#14 "renal failure" 4679

#15 MeSH descriptor: [Heart Failure] explode all trees 5854

#16 "heart failure" 14845

#17 "cardiac failure" 1242

#18 "myocardial failure" 27

#19 MeSH descriptor: [Pulmonary Disease, Chronic Obstructive] explode all trees 2689

#20 "chronic obstructive pulmonary disease" 5319

#21 "chronic obstructive lung disease" 1829

#22 "chronic airflow" next obstruct* 121

#23 "COPD" 8146

#24 "COAD" 143

#25 MeSH descriptor: [Diabetes Complications] explode all trees 4834

#26 MeSH descriptor: [Diabetes Mellitus] explode all trees 16993

#27 MeSH descriptor: [Diabetes Insipidus] explode all trees 43

#28 diabet* 43336

#29 MeSH descriptor: [Frail Elderly] explode all trees 534

#30 frail next elder* 862

#31 "frail older adult" 3

#32 MeSH descriptor: [Mortality] explode all trees 11330

#33 mortalit* 53183

#34 MeSH descriptor: [Risk Factors] explode all trees 20426

#35 high near risk 28077

#36 (#32 or #33) and (#34 and #35) 996

#37 MeSH descriptor: [Patient Readmission] explode all trees 833

#38 readmission 2611

#39 readmit* 407

#40 (#34 or #35) and (#37 or #38 or #39) 617

#41 {or #1-#31} or #36 or #40 86873

#42 virtual next ward* 1

#43 MeSH descriptor: [Telemedicine] explode all trees 1351

#44 telemedicine 1697

#45 "hospital home" 76

#46 "hospital at home" 111

#47 "home hospital" 73

#48 "remote consultation" 369

#49 teleconsultation 304

#50 "remote consultation" 369

#51 {or #43-#50} 2358

#52 MeSH descriptor: [Ambulatory Care] explode all trees 3512

#53 MeSH descriptor: [Patient-Specific Modeling] explode all trees 2

#54 "patient specific modeling" 2

#55 MeSH descriptor: [Case Management] explode all trees 665

#56 MeSH descriptor: [Monitoring, Physiologic] this term only 1941

#57 "physiological monitoring" 40

#58 "patient monitoring" 1502

#59 MeSH descriptor: [Patient Care Team] explode all trees 1456

#60 "patient care" next team* 1531

#61 healthcare next team* 99

#62 Health next team* 201

#63 {or #52-#62} 9161

#64 MeSH descriptor: [User-Computer Interface] this term only 955

#65 MeSH descriptor: [Internet] this term only 1939

#66 internet 6562

#67 web 7380

#68 virtual 1861

#69 MeSH descriptor: [Mobile Applications] explode all trees 17

#70 {or #64-#69} 13933

#71 #63 and #70 273

#72 #51 or #71 2566

#73 #41 and #72 767

**EMBASE:**

1. exp kidney failure/

2. renal insufficienc$.ti,ab,kw.

3. exp renal replacement therapy/

4. (kidney adj2 dialys?s).ti,ab,kw.

5. (renal adj2 dialys?s).ti,ab,kw.

6. end stage renal disease.ti,ab,kw.

7. end stage kidney disease.ti,ab,kw.

8. chronic kidney failure/

9. renal failure.ti,ab,kw.

10. kidney failure.ti,ab,kw.

11. hemodiafiltration/

12. h?emodialys?s.ti,ab,kw.

13. peritoneal dialys?s.ti,ab,kw.

14. extracorporeal dialys?s.ti,ab,kw.

15. exp heart failure/

16. heart failure.ti,ab,kw.

17. cardiac failure.ti,ab,kw.

18. myocardial failure.ti,ab,kw.

19. congestive heart failure.ti,ab,kw.

20. diastolic heart failure.ti,ab,kw.

21. systolic heart failure.ti,ab,kw.

22. chronic obstructive lung disease/

23. COPD.ti,ab,kw.

24. COAD.ti,ab,kw.

25. chronic obstructive pulmonary disease.ti,ab,kw.

26. chronic airflow obstruction.ti,ab,kw.

27. chronic obstructive lung disease.ti,ab,kw.

28. exp diabetes mellitus/

29. diabet$.ti,ab,kw.

30. exp diabetes insipidus/

31. frail elderly/

32. frail elder$.ti,ab,kw.

33. frail older adult$.ti,ab,kw.

34. mortality/

35. risk factor/

36. (high adj2 risk).ti,ab,kw.

37. 34 or 35 or 36

38. hospital readmission/

39. readmission.ti,ab,kw.

40. readmit$.ti,ab,kw.

41. 38 or 39 or 40

42. 37 and 41

43. 1 or 2 or 3 or 4 or 5 or 6 or 7 or 8 or 9 or 10 or 11 or 12 or 13 or 14 or 15 or 16 or 17 or 18 or 19 or 20 or 21 or 22 or 23 or 24 or 25 or 26 or 27 or 28 or 29 or 30 or 31 or 32 or 33 or 42

44. (virtual adj2 ward*).ti,ab,kw.

45. exp telemedicine/

46. mobile health.ti,ab,kw.

47. mhealth.ti,ab,kw.

48. (hospital adj2 home).ti,ab,kw.

49. remote consultation$.ti,ab,kw.

50. telehealth.ti,ab,kw.

51. or/44-50

52. exp ambulatory care/

53. patient specific modeling.ti,ab,kw.

54. case management/

55. exp physiologic monitoring/

56. (physiological adj2 monitor$).ti,ab,kw.

57. exp patient monitoring/

58. (patient adj2 monitor$).ti,ab,kw.

59. patient care/

60. patient care team$.ti,ab,kw.

61. (interdisciplinary adj2 care).ti,ab,kw.

62. interdisciplinary health.ti,ab,kw.

63. (collaborative adj2 care).ti,ab,kw.

64. (health adj2 team$).ti,ab,kw.

65. healthcare team$.ti,ab,kw.

66. or/52-65

67. computer interface/

68. internet/

69. internet.ti,ab,kw.

70. mobile application/

71. web.ti,ab,kw.

72. virtual.ti,ab,kw.

73. or/67-72

74. 66 and 73

75. 51 or 74

76. 43 and 75

77. randomized controlled trial/

78. controlled clinical trial/

79. randomization/

80. double blind procedure/

81. single blind procedure/

82. exp clinical trials/

83. (clin$ adj25 trial$).ti,ab.

84. ((singl$ or doubl$ or trebl$ or tripl$) adj25 (blind$ or mask$)).ti,ab.

85. placebo$.ti,ab.

86. random$.ti,ab.

87. methodology.sh.

88. comparative study/

89. exp evaluation studies/

90. follow up/

91. prospective study/

92. retrospective study/

93. (control$ or prospectiv$ or retrospectiv$ or volunteer$).ti,ab.

94. or/1-17

95. exp animals/ not humans/

96. 94 not 95

97. 76 and 96

CINAHL:

| S89 | S69 AND S88 |
| --- | --- |
| S88 | S87 NOT S86 |
| S87 | S70 OR S71 OR S72 OR S73 OR S74 OR S75 OR S76 OR S77 OR S78 OR S79 OR S80 OR S81 OR S82 OR S83 OR S84 OR S85 |
| S86 | TI ((MH "animals, laboratory") OR (MH "bats") OR (MH "cats") OR (MH "cattle") OR (MH "dogs") OR (MH "dolphins") OR (MH "Horses") OR (MH "primates") OR (MH "rabbits") OR (MH "Rodents") OR (MH "sheep") OR (MH "swine")) NOT (MH "Human") |
| S85 | TI ((singl* OR doubl* OR tripl* OR trebl*) N25 (blind* OR mask*)) |
| S84 | AB ((singl* OR doubl* OR tripl* OR trebl*) N25 (blind* OR mask*)) |
| S83 | AB random* |
| S82 | TI random* |
| S81 | TX "retrospective design" |
| S80 | TX "retrospective stud*" |
| S79 | MH "multicenter studies" |
| S78 | MH "evaluation research" OR (AB "evaluation stud*") OR (TI "evaluation stud*") |
| S77 | MH "prospective studies" OR (TI "prospective stud*") OR (AB "prospective stud*") |
| S76 | (MH "comparative studies") OR (TI "comparative stud*") OR (AB "comparative stud*) |
| S75 | MH "randomized controlled trials" |
| S74 | MH "intervention trials" |
| S73 | MH "triple-blind studies" |
| S72 | MH "single-blind studies" |
| S71 | MH "double-blind studies" |
| S70 | PT "clinical trial" |
| S69 | S39 AND S68 |
| S68 | S47 OR S67 |
| S67 | S59 AND S66 |
| S66 | S60 OR S61 OR S62 OR S63 OR S64 OR S65 |
| S65 | TX "virtual" |
| S64 | TX "remote" |
| S63 | TX "internet" |
| S62 | MH "world wide web" |
| S61 | MH "internet" |
| S60 | (MH "user-computer interface") OR (MH "mobile applications") |
| S59 | S48 OR S49 OR S50 OR S51 OR S52 OR S53 OR S54 OR S55 OR S56 OR S57 OR S58 |
| S58 | TX "healthcare team*" |
| S57 | TX "health team*" |
| S56 | TX "interdisciplinary healthcare" |
| S55 | TX "interdisciplinary care" |
| S54 | MH "case management" |
| S53 | TX "patient care team*" |
| S52 | MH "multidisciplinary care team*" |
| S51 | TX "patient monitoring" |
| S50 | MH "monitoring, physiologic" |
| S49 | TX "patient specific modeling" |
| S48 | MH "ambulatory care" |
| S47 | S40 OR S41 OR S42 OR S43 OR S44 OR S45 OR S46 |
| S46 | TX "telehealth" |
| S45 | TX "teleconsultation" |
| S44 | TX "hospital at home" |
| S43 | TX "home hospital" |
| S42 | TX "hospital home" |
| S41 | MH "telemedicine+" |
| S40 | TX virtual ward* |
| S39 | S1 OR S2 OR S3 OR S4 OR S5 OR S6 OR S7 OR S8 OR S9 OR S10 OR S11 OR S12 OR S13 OR S14 OR S15 OR S16 OR S17 OR S18 OR S19 OR S20 OR S21 OR S22 OR S23 OR S24 OR S25 OR S26 OR S36 OR S38 |
| S38 | S34 AND S37 |
| S37 | S27 OR S28 |
| S36 | (S31 OR S32 OR S33) AND (S34 AND S35) |
| S35 | S31 OR S32 OR S33 |
| S34 | S29 OR S30 |
| S33 | TX "readmit*" |
| S32 | TX "readmission" |
| S31 | MH "readmission" |
| S30 | TX "high* risk" |
| S29 | MH "risk factors" |
| S28 | TX "mortalit*" |
| S27 | MH "mortality" |
| S26 | TX "frail older adult" |
| S25 | TX "frail elder*" |
| S24 | MH "frail elderly" |
| S23 | TX "diabet*" |
| S22 | MH "diabetes mellitus+" |
| S21 | TX "COAD" |
| S20 | TX "COPD" |
| S19 | TX "chronic airflow obstruct*" |
| S18 | TX "chronic obstructive airway disease" |
| S17 | TX "chronic obstructive pulmonary disease" |
| S16 | MH "pulmonary disease, chronic obstructive" |
| S15 | TX "myocardial failure" |
| S14 | TX "cardiac failure" |
| S13 | MH "heart failure" |
| S12 | TX "extracorporeal dialys*" |
| S11 | TX "haemodialys*" |
| S10 | TX "hemodialys*" |
| S9 | TX "peritoneal dialys*" |
| S8 | TX "kidney dialys*" |
| S7 | TX "renal dialys*" |
| S6 | MH "renal replacement therapy+" |
| S5 | TX "kidney failure" |
| S4 | TX "end stage kidney diseas*" |
| S3 | TX "chronic renal diseas*" |
| S2 | TX "chronic kidney diseas*" |
| S1 | MH "renal insufficiency+" |

**SCOPUS:**

| TITLE-ABS-KEY ("renal insufficienc*") |
| --- |
| TITLE-ABS-KEY (renal W/2 replacement W/2 therap* ) |
| TITLE-ABS-KEY (renal W/2 dialys*s ) |
| TITLE-ABS-KEY (kidney W/2 dialys*s ) |
| TITLE-ABS-KEY (peritoneal W/2 dialys*s ) |
| TITLE-ABS-KEY (h*emodial*s*s ) |
| TITLE-ABS-KEY (extracorporeal W/2 dialys*s ) |
| TITLE-ABS-KEY (kidney W/2 disease* ) |
| TITLE-ABS-KEY ("chronic kidney disease*" ) |
| TITLE-ABS-KEY ("chronic renal disease*" ) |
| TITLE-ABS-KEY ("end-stage kidney disease* ") |
| TITLE-ABS-KEY ("kidney failure") |
| TITLE-ABS-KEY ("renal failure") |
| TITLE-ABS-KEY ("heart failure") |
| TITLE-ABS-KEY ("cardiac failure") |
| TITLE-ABS-KEY ("myocardial failure") |
| TITLE-ABS-KEY (COPD) |
| TITLE-ABS-KEY ("chronic obstructive pulmonary disease") |
| TITLE-ABS-KEY (COAD) |
| TITLE-ABS-KEY ("chronic airflow obstruction") |
| TITLE-ABS-KEY ("chronic obstructive lung disease") |
| TITLE-ABS-KEY (diabet*) |
| TITLE-ABS-KEY ("frail elder*") |
| TITLE-ABS-KEY ("frail older adult*") |
| TITLE-ABS-KEY (mortality) |
| TITLE-ABS-KEY ("risk factor*") |
| TITLE-ABS-KEY (high W/2 risk) |
| 25 or 26 or 27 |
| TITLE-ABS-KEY (readmission) |
| TITLE-ABS-KEY (readmit*) |
| 29 or 30 |
| 28 and 31 |
| 1 or 2 or 3 or 4 or 5 or 6 or 7 |
| 8 or 9 or 10 or 11 or 12 or 13 or 14 |
| 15 or 16 or 17 or 18 or 19 or 20 or 21 |
| 22 or 23 or 24 or 32 |
| 33 or 34 or 35 or 36 |
| TITLE-ABS-KEY (virtual W/2 ward*) |
| TITLE-ABS-KEY (telemedicine) |
| TITLE-ABS-KEY ("mobile health") |
| TITLE-ABS-KEY (mhealth) |
| TITLE-ABS-KEY (hospital W/2 home) |
| TITLE-ABS-KEY ("remote consultation") |
| TITLE-ABS-KEY (teleconsultation) |
| TITLE-ABS-KEY (telehealth) |
| 38 or 39 or 40 or 41 or 42 or 43 or 44 |
| 45 or 46 |
| TITLE-ABS-KEY ("ambulatory care") |
| TITLE-ABS-KEY ("patient specific monitoring") |
| TITLE-ABS-KEY ("case management") |
| TITLE-ABS-KEY ("physiologic* monitoring") |
| TITLE-ABS-KEY ("patient monitoring") |
| TITLE-ABS-KEY ("patient care team*") |
| TITLE-ABS-KEY (interdisciplinary W/2 care) |
| TITLE-ABS-KEY ("interdisciplinary health") |
| TITLE-ABS-KEY (collaborative W/2 care) |
| TITLE-ABS-KEY (health W/2 team*) |
| TITLE-ABS-KEY ("healthcare team*") |
| 48 or 49 or 50 or 51 or 52 or 53 |
| 54 or 55 or 56 or 57 or 58 |
| 59 or 60 |
| TITLE-ABS-KEY ("user-computer interface") |
| TITLE-ABS-KEY (internet) |
| TITLE-ABS-KEY (web) |
| TITLE-ABS-KEY (virtual) |
| TITLE-ABS-KEY ("mobile application*") |
| 62 or 63 or 64 or 65 or 66 |
| 61 and 67 |
| 47 or 68 |
| 37 and 69 |
| TITLE-ABS-KEY ("randomized controlled trial*") |
| TITLE-ABS-KEY ("controlled clinical trial*") |
| TITLE-ABS-KEY ("random allocation") |
| TITLE-ABS-KEY ("double-blind method") |
| TITLE-ABS-KEY ("single-blind method") |
| 71 or 72 or 73 or 74 or 75 |
| TITLE-ABS (clin* W/25 trial*) |
| TITLE-ABS ((singl* or doubl* or trebl* or tripl*) W/25 (blind* or mask*)) |
| TITLE-ABS-KEY (placebo*) |
| TITLE-ABS (random*) |
| 77 or 78 or 79 or 80 |
| TITLE-ABS-KEY ("comparative stud*") |
| TITLE-ABS-KEY ("evaluation stud*") |
| TITLE-ABS-KEY ("follow up stud*") |
| TITLE-ABS-KEY ("prospective stud*") |
| TITLE-ABS-KEY ("retrospective stud*") |
| TITLE-ABS(control* or prospectiv* or retrospectiv* or volunteer*) |
| 82 or 83 or 84 or 85 or 86 or 87 |
| 76 or 81 or 88 |
| 70 and 89 |
| INDEXTERMS(animal*) AND NOT INDEXTERMS(human*) |
| 90 AND NOT 91 |
| ((PUBYEAR < 2017) OR (PUBDATETXT(January 2017))) |
| 92 and 93 |
